# Author's Corrigendum: Hemopoiesis in the Thymus

**DOI:** 10.1155/1995/12606

**Published:** 1995

**Authors:** Marion D. Kendall

**Affiliations:** 1 Thymus Laboratory The Babraham Institute Cambridge CB2 4AT United Kingdom; 2 Pharmacology Department UMDS London SE1 7EH United Kingdom


					Developmental Immunology, 1996, Vol 4, pp. 317
Reprints available directly from the publisher
Photocopying permitted by license only
(C) 1996 OPA (Overseas Publishers Association)
Amsterdam B.V. Published in The Netherlands
by Harwood Academic Publishers GmbH
Printed in Singapore
Author's Corrigendum
Hemopoiesis in the Thymus
MARION D. KENDALL
Thymus Laboratory, The Babraham Institute, Cambridge CB2 4AT, and Pharmacology Department, UMDS, London SE1 7EH, UK
The above paper (Vol. 4. No. 3, pp. 157-168) was published with the omission of a sentence. The
omission occurred on the sixth line from the end of page 157. The information in the following
paragraph is correct.
mouse has two lineages of stem cells. The majority were Thyl1, Lin-, Sca-1 cells, with potent myeloerythroid
lineage capabilities as well as being thymus-repopulating cells. However, a small population of similar cells
but Sca- also had CFU-S (colony forming units-spleen) capabilities and were probably on the erythroid line
of development.Alternatively; truly multi-potential stem cells of the embryo might remain in the thymus in
adult life, and on occasion be "awakened". Indeed, variations on the extent to which this might happen
could be the key to many of the observations quoted in this review.

				

